# Preoperative malnutrition with mild hypoalbuminemia associated with postoperative mortality and morbidity of colorectal cancer: a propensity score matching study

**DOI:** 10.1186/s12937-019-0458-y

**Published:** 2019-06-28

**Authors:** Wan-Hsiang Hu, Samuel Eisenstein, Lisa Parry, Sonia Ramamoorthy

**Affiliations:** 1 Department of Colorectal Surgery, Kaohsiung Chang Gung Memorial Hospital, Chang Gung University College of Medicine, Kaohsiung, Taiwan; 2 Department of Surgery, University of California, San Diego Health System, La Jolla, San Diego, CA USA; 3 Department of Surgery and Rebecca and John Moores Cancer Center, University of California San Diego, 3855 Health Sciences Drive, La Jolla, San Diego, CA 92093 USA

**Keywords:** Mild hypoalbuminemia, Postoperative mortality and morbidity, Colorectal cancer, Propensity score matching

## Abstract

**Background:**

Malnutrition with hypoalbuminemia (albumin < 35 g/L) is an important factor in predicting risks associated with colorectal cancer surgery. However, there is limited data about the effects of mild hypoalbuminemia with small decreases in albumin on postoperative complications.

**Methods:**

This is a retrospective study using the multi-institutional, nationally validated database of the American College of Surgeons-National Surgical Quality Improvement Program (ACS-NSQIP) to investigate mild hypoalbuminemia and its association with postoperative mortality and morbidity by using a propensity score matching method.

**Results:**

In a group of 30,676 colorectal cancer patients who received surgery, 5230 had mild hypoalbuminemia (< 35 and > =30 g/L) and 21,310 had normal albumin levels (> = 35 g/L). Significant differences were noted in 21 clinical characteristics between the two groups. After 1:2 propensity score matching postoperative mortality was significantly associated with mild hypoalbuminemia (OR = 1.74; *p* < 0.001). There were significant associations between mild hypoalbuminemia and 11 postoperative morbidities including deep vein thrombosis, pulmonary embolism, superficial and deep surgical site infection, pneumonia, septic shock, ventilator> 48 h, blood transfusion, return to operating room, stroke and re-intubation. Mild hypoalbuminemia was also associated with overall complication (B = 0.064, *p* < 0.001) and length of total hospital stay (B = 2.236, *p* < 0.001).

**Conclusions:**

In colorectal cancer, this is the first propensity score matching study of malnutrition with mild hypoalbuminemia which demonstrates that a mild decrease in serum albumin contributes significantly to poor postoperative outcome.

**Electronic supplementary material:**

The online version of this article (10.1186/s12937-019-0458-y) contains supplementary material, which is available to authorized users.

## Background

Malnutrition is a significant risk factor in postoperative mortality, morbidities and length of hospital stay in hospitalized [1], surgical [2] and cancer patients [3]. Preoperative serum albumin serves as an excellent tool to assess malnutrition and to predict patient outcomes and survival [4–10]. Detection of hypoalbuminemia and appropriate intervention could decrease the complication rates [11] when associated with malnutrition.

In the United States, colorectal cancer is the third most common cancer [12] and it is the most common cancer in Taiwan [13]. Our previous study and other studies in the literature showed that malnutrition with hypoalbuminemia (< 35 g/L) is a predominant problem in colorectal cancer and is associated with poor postoperative outcomes [14–16]. Serum albumin is a negative acute-phase protein with reduced expression and increased losses during inflammation processes [17] and in underweight people. Therefore hypoalbminemia is an indicator for malnutrition in acute and chronically ill patients. However, some studies concluded that hypoalbuminemia only becomes clinically significant at levels < 25 g/L [18, 19], and that albumin replacement is only covered by health insurance in Taiwan for patients with serum albumin levels < 30 g/L and who exhibit associated comorbidities. Subgroup analyses of the association between a mild decrease of serum albumin (< 35 and > =30 g/L) and postoperative complications is rare and generally focused on procedures [20]. We wanted to study the effect of mild hypoalbuminemia on the postoperative outcomes in colorectal cancer patients.

The American College of Surgeons-National Surgical Quality Improvement Program (ACS-NSQIP) database records the preoperative comorbidities and postoperative complications of many kinds of operations from more than five hundred medical hospitals in the United States and Canada [21]. Propensity score matching provides a method to minimize bias from observational treatment cohorts and seeks to approximate the characteristics of a randomized clinical trial [22]. The purpose of this study was to apply propensity score matching to this robust database to compare the postoperative outcomes in colorectal cancer patients with mild hypoalbuminemia to those with normal serum albumin.

## Methods

### Patient selection

Data of the ACS-NSQIP from 2009 to 2013 was utilized to identify colorectal cancer patients using ICD-9 (International Classification of Disease, Ninth Revision) diagnosis codes (Additional file 1). Colorectal cancer patients undergoing related operations were selected by the Current Procedural Terminology (CPT) codes (Additional file 1) in: principle operative procedure; other procedure; or concurrent procedure records.

### Postoperative outcomes

The primary outcomes studied were 30-day mortality, all morbidities, overall complication and length of total hospital stay. The postoperative morbidities included: surgical site infection (superficial, deep and organic); wound disruption; urinary tract infection; pneumonia; re-intubation; ventilator> 48 h; pulmonary embolism; deep vein thrombosis; progressive renal insufficiency; acute renal failure; stroke; myocardial infarction; cardiopulmonary resuscitation; blood transfusion; sepsis; septic shock; and return to operating room. Mortality and morbidities were graded and weighted using the Accordion Severity Grading System [23, 24]. The overall complication score was calculated as the sum of the weighted scores in each patient.

### Statistical analysis

For our analyses, “control” was defined by a normal serum albumin levels (> = 35 g/L), and “case” was defined by mild hypoalbuminemia (< 35 and > =30 g/L) as measured by laboratory data collected within 2 weeks before surgery. Individual propensity scores were calculated by logistic regression method based on 21 clinical associated factors including age, gender, body mass index, smoking, emergent operation and other comorbidities. A 1:2 ratio propensity score matching study group was created using the Greedy method with a 0.2 caliper width using NCSS 10 software (NCSS Statistical Software, Kaysville, UT, USA) [25]. A chi-square test was used for univariate association. Binary logistic regression method was used to analyze the association between mild hypoalbuminemia and postoperative mortality and morbidity. The association among length of total hospital stay, overall morbidity and mild hypoalbuminemia was analyzed with regression analysis. Tests were two-tailed and statistical significance was defined at *p* < 0.05. All statistical analyses were performed on SPSS for Windows version 22.

## Results

From the database of the ACS-NSQIP during the years 2009 to 2013, a total of 30,676 colorectal cancer patients who received associated surgery were identified. We selected 5230 (17%) with mild hypoalbuminemia (< 35 and > =30 g/L) and 21,310 (70%) with normal serum albumin levels (> = 35 g/L) for further study. Significant differences with respect to 21 clinical characteristics were demonstrated between the two groups (Table 1). The patients with mild hypoalbuminemia were older, more likely to be thin, female, used tobacco and steroids more frequently, had more comorbidities, and higher grades of American Society of Anesthesiologists and wound classification. After 1:2 ratio propensity score matching, 4305 patients with mild hypoalbuminemia and 8610 normal patients were retained for comparison. No significant differences in previously associated covariates were noted between the two groups (Table 1).

**Table 1:** Selected Cohort Characteristics Before and After Propensity Score-Matching

Variables	Full Cohort			Matched Cohort (1:2)		
	Albumin (g/dl)		*P* value	Albumin (g/dl)		*P* value
	< 3.5 and > =3.0 (*n* = 5230)	> = 3.5 (*n* = 21,310)		< 3.5 and > =3.0 (*n* = 4305)	> = 3.5 (*n* = 8610)	
Age, mean (SD), y	70.2(13.7)	65.2(13.6)	< 0.001	68.9(13.8)	69.3(13.1)	0.075
BMI, mean (SD)	27.6(7.2)	28.2(6.5)	< 0.001	28(7.2)	27.9(6.3)	0.441
Gender, male, n(%)	2527(48.3)	11,370(53.4)	< 0.001	2140 (49.7)	4290(49.8)	0.901
Smoking, n(%)	834(15.9)	3125(14.7)	0.02	677(15.7)	1327(15.4)	0.643
Ventilator, n(%)	6(0.1)	12(0.05)	0.146	4(0.09)	3(0.03)	0.181
COPD, n(%)	469(9)	1061(5)	< 0.001	325(7.5)	607(7)	0.301
Ascites, n(%)	108(2.1)	128(0.6)	< 0.001	43(1)	60(0.7)	0.069
CHF, n(%)	137(2.6)	161(0.8)	< 0.001	42(1)	74(0.9)	0.51
Hypertension, n(%)	3104(59.3)	11,492(53.9)	< 0.001	2517(58.5)	5072(58.9)	0.631
Renal failure, n(%)	13(0.2)	27(0.1)	0.042	4(0.1)	15(0.2)	0.256
Dialysis, n(%)	47(0.9)	80(0.4)	< 0.001	21(0.5)	48(0.6)	0.609
DC, n(%)	770(14.7)	1977(9.3)	< 0.001	539(12.5)	1048(12.2)	0.57
Steroid, n(%)	185(3.5)	541(2.5)	< 0.001	140(3.3)	259(3)	0.45
Emergency, n(%)	503(9.6)	834(3.9)	< 0.001	242(5.6)	429(5)	0.123
BWL, n(%)	606(11.6)	1007(4.7)	< 0.001	315(7.3)	574(6.7)	0.169
Diabetes mellitus, n(%)			< 0.001			0.679
No	4083(78.1)	17,604(82.6)		3415(79.3)	6816(79.1)	
Oral	723(13.8)	2702(12.7)		607(14.1)	1194(13.9)	
Insulin	424(8.1)	1004(4.7)		283(6.6)	600(7)	
Dyspnea			< 0.001			0.590
No	4443(85)	19,340(90.8)		3753(87.2)	7546(87.7)	
Exertion	696(13.3)	1858(8.7)		515(12)	1002(11.6)	
Rest	91(1.7)	112(0.5)		37(0.8)	62(0.7)	
Systemic sepsis			< 0.001			0.156
No	4798(91.7)	20,775(97.5)		4148(96.4)	8355(97)	
SIRS	303(5.8)	386(1.8)		114(2.6)	188(2.2)	
Sepsis	118(2.3)	128(0.6)		39(0.9)	57(0.7)	
Septic shock	11(0.2)	21(0.1)		4(0.1)	10(0.1)	
Functional status^a^, n(%)			< 0.001			0.883
Independent	4781(91.4)	20,749(97.4)		4154(96.5)	8322(96.6)	
Partially Dependent	381(7.3)	504(2.4)		140(3.2)	266(3.1)	
Totally Dependent	68(1.3)	57(0.3)		11(0.3)	22(0.3)	
ASA			< 0.001			0.169
I	42(0.8)	519(2.4)		42(1)	95(1.1)	
II	1256(24)	8779(41.2)		1213(28.2)	2273(26.4)	
III	3307(63.2)	11,009(51.7)		2753(63.9)	5634(65.44)	
IV	620(11.9)	996(4.67)		295(6.85)	607(7.05)	
V	5(0.1)	7(0.03)		2(0.05)	1(0.01)	
Wound			< 0.001			0.343
II	4526(86.5)	19,604(92)		3885(90.24)	7805(90.6)	
III	375(7.2)	1252(5.9)		285(6.62)	574(6.7)	
IV	329(6.3)	454(2.1)		135(3.14)	231(2.7)	

*BMI* body mass index, *COPD* chronic obstructive pulmonary disease, *CHF* Congestive heart failure, *DC* Disseminated cancer, *BWL* body weight loss, *ASA* American Society of Anesthesiologists, *Wound II* clean/contamination, *Wound III* contamination, *Wound IV* dirty

^a^ assistance from another person for any activities of daily living

Table 2 shows the difference of rates of postoperative outcomes between normal and mild hypoalbuminemia patients before matching. Mortality and all morbidities, except organic surgical site infection, were significantly associated with mild hypoalbuminemia. After matching, the patients with mild hypoalbuminemia still had significantly higher in-hospital mortality rates compared with those with normal albumin levels (2.3% vs. 1.3%; OR = 1.74; *p* < 0.001) (Table 3). Low serum albumin had significant association with deep vein thrombosis (OR = 1.99; *p* < 0.001) and pulmonary embolism (OR = 1.55; *p* = 0.011). Problems related with infection including superficial surgical site infection, deep surgical site infection, pneumonia and septic shock occurred in patients with mild hypoalbuminemia. The other significant postoperative outcomes included ventilator dependence more than 48 h, blood transfusion, return to operating room, stroke and re-intubation. Higher incidence of other postoperative complications, like wound disruption, were noted in the mild hypoalbuminemia patients.

**Table 2:** The association of postoperative mortality and morbidity in patients grouped by albumin level before propensity score matching

Postop outcomes	Albumin (g/dl)		OR (95% CI)	*P* value
	< 3.5 and > =3.0 (*n* = 5230)	> = 3.5 (*n* = 21,310)		
30-day mortality	196(3.7)	248(1.2)	3.31(2.74–4.00)	< 0.001
Stroke	40(0.8)	55(0.3)	2.98(1.98–4.48)	< 0.001
DVT	142(2.7)	239(1.1)	2.46(2.00–3.04)	< 0.001
Ventilator> 48 h	183(3.5)	319(1.5)	2.39(1.98–2.87)	< 0.001
Septic shock	159(3)	280(1.3)	2.36(1.93–2.87)	< 0.001
Transfusion	991(18.9)	2009(9.4)	2.25(2.07–2.44)	< 0.001
Re-intubation	177(3.4)	365(1.7)	2.01(1.68–2.41)	< 0.001
Pneumonia	211(4)	457(2.1)	1.92(1.63–2.27)	< 0.001
PRI	70(1.3)	163(0.8)	1.76(1.33–2.33)	< 0.001
Deep SSI	113(2.2)	302(1.4)	1.54(1.24–1.91)	< 0.001
Sepsis	245(4.7)	715(3.4)	1.42(1.22–1.64)	< 0.001
UTI	217(4.1)	646(3)	1.39(1.18–1.62)	< 0.001
Return to OR	353(6.7)	1068(5)	1.37(1.21–1.55)	< 0.001
Superficial SSI	430(8.2)	1411(6.6)	1.26(1.13–1.41)	< 0.001
ARF	49(0.9)	116(0.5)	1.73(1.24–2.42)	0.001
PE	71(1.4)	180(0.8)	1.62(1.23–2.13)	0.001
CPR	44(0.8)	103(0.5)	1.75(1.23–2.49)	0.002
MI	63(1.2)	163(0.8)	1.58(1.18–2.12)	0.002
Wound disruption	85(1.6)	259(1.2)	1.34(1.05–1.72)	0.019
Organ SSI	216(4.1)	869(4.1)	1.01(0.87–1.18)	0.865

Values in parentheses are percentage

*DVT* deep vein thrombosis, *PRI* progressive renal insufficiency, *SSI* surgical site infection, *UTI* urinary tract infection, *OR* operating room, *ARF* acute renal failure, *PE* pulmonary embolism, *CPR* cardiopulmonary resuscitation, *MI* myocardial infarction

**Table 3:** The association of postoperative mortality and morbidity in patients grouped by albumin level after propensity score matching

Postop outcomes	Albumin (g/dl)		OR (95% CI)	*P* value
	< 3.5 and > =3.0 (*n* = 4305)	> = 3.5 (*n* = 8610)		
30-day mortality	98(2.3)	114(1.3)	1.74(1.32–2.28)	< 0.001
DVT	109(2.5)	111(1.3)	1.99(1.52–2.60)	< 0.001
Ventilator> 48 h	113(2.6)	145(1.7)	1.57(1.23–2.02)	< 0.001
Transfusion	737(17.1)	991(11.5)	1.59(1.43–1.76)	< 0.001
Return to OR	291(6.8)	447(5.2)	1.32(1.14–1.54)	< 0.001
Superficial SSI	367(8.5)	599(7)	1.25(1.09–1.42)	0.001
Pneumonia	151(3.5)	214(2.5)	1.43(1.15–1.76)	0.001
Septic shock	100(2.3)	133(1.5)	1.52(1.17–1.97)	0.002
Deep SSI	98(2.3)	138(1.6)	1.43(1.10–1.86)	0.007
PE	60(1.4)	78(0.9)	1.55(1.10–2.17)	0.011
Stroke	27(0.6)	28(0.3)	1.93(1.14–3.29)	0.013
Re-intubation	124(2.9)	193(2.2)	1.29(1.03–1.63)	0.027
Sepsis	189(4.4)	320(3.7)	1.19(0.99–1.43)	0.064
UTI	173(4)	304(3.5)	1.14(0.95–1.38)	0.166
Wound disruption	69(1.6)	116(1.3)	1.19(0.88–1.61)	0.249
PRI	47(1.1)	76(0.9)	1.24(0.86–1.79)	0.249
ARF	31(0.7)	48(0.6)	1.29(0.82–2.04)	0.264
MI	50(1.2)	91(1.1)	1.10(0.78–1.56)	0.590
Organ SSI	176(4.1)	366(4.3)	0.96(0.80–1.15)	0.664
CPR	26(0.6)	51(0.6)	1.02(0.64–1.64)	0.936

Values in parentheses are percentage

*DVT* deep vein thrombosis, *OR* operating room, *SSI* surgical site infection, *PE* pulmonary embolism, *PRI* progressive renal insufficiency, *ARF* acute renal failure, *UTI* urinary tract infection, *CPR* cardiopulmonary resuscitation, *MI* myocardial infarction

Regression analyses revealed that overall complication and length of total hospital stay were associated with mild hypoalbuminemia. After matching, both overall complication (B = 0.064, *p* < 0.001) and length of total hospital stay (B = 2.236, *p* < 0.001) were still greater in the mild hypoalbuminemia group than in the group with normal serum albumin levels (Table 4).

**Table 4:** Regression analysis of overall complication and length of total hospital stay in patients grouped by albumin level before and after propensity score matching

Post-operative outcomes	Albumin (g/dl)	Mean	B (coefficient)	95% C.I.	*P* value
Overall Complication^b^	< 3.5 and > =3.0	0.2821	0.121	0.107–0.135	< 0.001
	> = 3.5	0.1612	0	0	
Length of total hospital stay^b^	< 3.5 and > =3.0	11.0069	3.453	3.201–3.705	< 0.001
	> = 3.5	7.5539	0	0	
Overall Complication^a^	< 3.5 and > =3.0	0.2468	0.064	0.047–0.082	< 0.001
	> = 3.5	0.1826	0	0	
Length of total hospital stay^a^	< 3.5 and > =3.0	10.4407	2.236	1.902–2.57	< 0.001
	> = 3.5	8.2045	0	0	

^b^ before; ^a^ after

## Discussion

Using the nationally validated ACS-NSQIP database, our multi-institutional study demonstrates the effects of mild hypoalbuminemia on postoperative outcomes of colorectal cancer patients. We matched mild hypoalbuminemia patients and patients with normal serum albumin levels using a propensity score matching method to decrease the biases associated with the selecting of two groups with significant differences in other clinical characteristics. After matching, postoperative mortality, overall complication and length of hospital stay were still associated with mild hypoalbuminemia.

The association between hypoalbuminemia and comorbidities has been discussed in the literature. Hospitalized patients with mild hypoalbuminemia (albumin 25–35 g/L) were older, lower body mass index, and more hypertension, congestive heart failure and chronic renal failure than those with normal albumin levels [9]. Severe hypoalbuminemia was noted in 4% of chronic obstructive pulmonary disease patient and was a strong risk factor for developing acute renal failure [26]. The rates of hypoalbuminemia ranged from 20 to 25% in chronic heart failure patients to 90% in weak, elderly patients suffering from acute heart failure [27]. In our study, mild hypoalbuminemia was not rare and was significantly associated with many comorbidities in colorectal cancer patients. Because there is more postoperative risk associated with even mild hypoalbuminemia, early identification of malnutrition in patients with multiple comorbidities is recommended.

In our retrospective study, randomization was not possible. However, strong associations between mild hypoalbuminemia and the patients’ clinical characteristics were demonstrated. We used a propensity score matching method to reduce the bias in estimating the effect of mild hypoalbuminemia on postoperative outcomes, and the likelihood of confounding when analyzing the observational data [28]. After matching, a mild decrease in serum albumin level still had serious effects on postoperative outcomes including increased mortality, morbidities, overall complication and length of total hospital stay.

After matching, venous thromboembolism (VTE), including deep vein thrombosis (OR = 1.99) and pulmonary embolism (OR = 1.55) was associated with mild hypoalbuminemia in our study. Aaron et al. [29] demonstrated low serum albumin was a modest marker of an increased risk of venous thromboembolism after adjusting for age, sex, race, use of hormone replacement therapy, estimated GFR, history of cancer, and diabetes. The adjusted hazard ratio for albumin below the fifth percentile was 1.28 and 1.8 in their two cohorts’ data. Hypoalbuminemia (serum albumin level < 35 mg/L) was reported to be associated with deep vein thrombosis (AOR 1.69) in colon and rectal surgery [30]. In cancer patients, a similar result was also noted [31]. The mechanism of association between hypoalbuminemia and VTE is poorly understood but may be due to hyperinflammatory or hypercoagulable states. Further studies of the mechanism are necessary, and closer monitoring for VTE in cancer patients with hypoalbuminemia is recommended.

A multi-institutional study showed preoperative hypoalbuminemia (serum albumin < 30 g/L) was an independent predictor for development of superficial and deep surgical site infection and prolonged hospital stay following gastrointestinal surgery [32]. The associations were also reported in other operative procedures [33–35]. In our study, a mild decrease in serum albumin also had a significant effect on surgical site infection, pneumonia, septic shock and length of total hospital stay. Low serum albumin was an excellent assessment tool for malnutrition [14], which was associated with poor wound healing [36] and infection [37]. Albumin serves an immunomodulatory role [38], and impairment of macrophage activation and granuloma formation were noted in hypoalbuminemic mice [39]. Serum albumin is a negative acute-phase protein during inflammatory reaction by bacterial infection [40]. These factors may promote surgical site infection and pneumonia in hypoalbuminemia and in mild hypoalbuminemic colorectal cancer patients.

There are several limitations to our study. First, the database only records the events that happened during the 30-day postoperative period, so it may not correctly estimate the true rate of postoperative outcomes, some of which may have occurred after 30 days. Second, nutritional supplementation may have been given to mild hypoalbuminemia patients and the information was not recorded. This means that part of the patients with mild hypoalbuminemia might receive supplementation pre- and postoperatively and their outcome was supposedly improved. The data of patients being purely without nutrition supplementation would make the results, ie. the mild hypoalbuminemia associated with worse outcome more contrasting. In addition, some of the important postoperative complications associated with colorectal surgery, like anastomotic leakage and ileus were not included in the database. Finally, the database has no cancer-specific variables like stage and tumor size, which may interact with mild hypoalbuminemia in postoperative outcome evaluation. We excluded the patients who only received palliative surgery to minimize the effect of the advanced stage on postoperative complications.

## Conclusions

In colorectal cancer patients, malnutrition with mild hypoalbuminemia is a common problem and associated with more comorbidities. After propensity score matching, a significant association with many postoperative complications was demonstrated. Randomized controlled trials are needed to evaluate if early identification and aggressive nutritional intervention in patients with even mild hypoalbuminemia would reduce the rate of postoperative complications and improve postoperative outcomes.

## Additional file

Additional file 1: ICD-9 code and CPT code. (DOCX 50 kb)

## Data Availability

Not applicable.

## Abbreviations

ACS-NSQIP: American College of Surgeons-National Surgical Quality Improvement Program
CPT: Current Procedural Terminology
ICD-9: International Classification of Disease, Ninth Revision
