# Supplementary material for: Preoperative malnutrition with mild hypoalbuminemia associated with postoperative mortality and morbidity of colorectal cancer: a propensity score matching study
Source: Nutr J. 2019 Jun 28;18:33. doi: 10.1186/s12937-019-0458-y (PMC6598281; doi:10.1186/s12937-019-0458-y)
Supplement: Supplementary file 1 — ICD-9 code and CPT code. (DOCX 50 kb) [file 12937_2019_458_MOESM1_ESM.docx]

ICD-9 code of colorectal cancer: 153-154.1 (exclude 153.5), 154.8 and 197.5

CPT code for colectomy and proctectomy in colorectal cancer

1. 44140-44147, 44160: partial colectomy, laparotomy
2. 44150-44158: total colectomy, laparotomy
3. 44204-44208: partial colectomy, laparoscopy
4. 44210-44212: total colectomy, laparoscopy
5. 45110: abdominoperineal resection
6. 45395: abdominoperineal resection, laparoscopy
7. 45111-45114, 45119: proctectomy, laparotomy
8. 45397: proctectomy, laparoscopy
